# Functional Constipation and Constipation-Predominant Irritable Bowel Syndrome in the General Population: Data from the GECCO Study

**DOI:** 10.1155/2016/3186016

**Published:** 2015-12-31

**Authors:** Paul Enck, Johannes Leinert, Menno Smid, Thorsten Köhler, Juliane Schwille-Kiuntke

**Affiliations:** ^1^Department of Internal Medicine VI, University Hospital Tübingen, 72076 Tuebingen, Germany; ^2^infas Institut, 53113 Bonn, Germany

## Abstract

*Background*. The prevalence of constipation in the (German) population has been shown to be 14.9% in a telephone survey, but more detailed data are required to characterize the sociographics and clinical characteristics of persons with different types of functional constipation, either constipation-predominant irritable bowel syndrome (IBS-C) or functional constipation with or without meeting Rome criteria.* Methods*. Of 2239 constipated individuals identified during the telephone interview, 1037 (46.3%) were willing to provide a postal address for a questionnaire, of which 589 (56.8%) returned the questionnaire, inquiring about sociographic data, clinical symptoms, and health care behavior related to constipation, as well as health-related quality-of-life (SF12). Subgroups of functionally constipated individuals were compared.* Results*. More than 50% of the respondents reported a somatic comorbid condition and/or regular medication intake that may contribute to constipation. We split the remaining individuals (*N* = 214) into three groups, matching Rome-criteria for IBS (IBS-C, *n* = 64) and for functional constipation (FC-R, *n* = 36) and FC not matching Rome criteria (*n* = 114). Nearly all sociographic and clinical characteristics were equal among them, and all individuals with constipation had similar and lowered QOL on the SF-12 physical health domain, but in IBS-C the scores were also significantly lower in comparison to FC-R and FC, in both the physical health and the mental health domain.* Conclusion*. Only a fraction of individuals with chronic constipation match Rome criteria for IBS-C or FC, but subgroups do not differ with respect to most other measures except quality-of-life profiles.

## 1. Background

Prevalence of chronic constipation has been reported to range between 5% and 15% of the general population, depending on the size and type of assessment, the definition of constipation, and variables such as nationality, culture, and the health care system [1–3]. In a recent paper we reported constipation to be 14.9% [4] in a representative sample from Germany, which is almost identical with the 14% reported pooled prevalence across 41 studies worldwide [5].

Constipation-predominant IBS (IBS-C) represents a subgroup of all IBS patients in whom abdominal pain is associated with constipation, while other predominant bowel symptoms (diarrhea, alternating diarrhea, and constipation) are labeled as IBS-D and IBS-M [6] when matching Rome criteria for IBS definitions [7]. Another Rome-defined functional bowel disorder with constipation as predominant symptom is “functional constipation” (FC-R). This definition requires not classifying for IBS-C, and at least 2 of six symptoms (need for straining, lumpy or hard stools, sensation of incomplete evacuation, sensation of anorectal obstruction, and need for manual maneuvers to facilitate defecation more than occasionally) or less than 3 defecations/week. Since constipation is (at least in epidemiological surveys) symptom-based and self-defined, there may as well be a group of people that perceive themselves as being constipated without matching Rome or other criteria [7], for example, reporting no abdominal pain or none of the additionally required symptoms. All these subgroups may be labeled “functional constipation”; their differential clinical phenotypes have only occasionally been described.

When patients with IBS-C were compared to patients with functional constipation matching or not matching Rome criteria, Rey et al. [6] found patients with painful or painless constipation not matching IBS-C criteria to be moderately younger and more frequently consulters in comparison to IBS-C, while Zhao et al. [8] comparing IBS-C to functional non-IBS constipation showed IBS-C to have a lesser need to strain but more incomplete evacuations after defection. Only the mental health subscale of the SF-36 was significantly lowered in IBS-C. Some differences were found with respect to upper GI symptoms: Koloski et al. [9] compared patients meeting Rome criteria for functional to patients with IBS-C and found only higher age at constipation onset, less likelihood to exercise, and higher mental health compared to IBS-C. Finally, Heidelbaugh et al. [10] compared patients with chronic idiopathic constipation with or without abdominal pain and IBS-C patients and found both IBS-C and chronic idiopathic constipation patients with pain significantly more bothered than constipation patients without pain.

Constipation may also occur as a secondary symptom, for example, in a number of neurological (e.g., stroke, Parkinson's disease, and spinal cord injury), systemic (diabetes, hypothyroidism, and scleroderma), and other disorders, to intestinal or nonintestinal surgery [11–13] or to a variety of medications used for treatment of chronic clinical conditions, for example, calcium antagonists for high blood pressure [14], opioids for chronic pain [15], and tricyclic antidepressants for major depression [16].

Quality-of-life (QoL) has been reported to be low in functional constipation as well as in IBS-C when assessed with both constipation-specific QoL instruments [17] and generic QoL tests in comparison to other chronic conditions [18, 19]; however they have yet not been compared between subgroups (except [6, 8]).

The purpose of the present evaluation of the German Chronic Constipation (GECCO) study data was to compare different populations with functional constipation with or without abdominal pain with respect to sociographic, clinical, and quality-of-life data. We assumed representativeness of our samples allowing conclusions on the population prevalence of the different subgroups but tested for response biases beforehand.

## 2. Methods

The aim of GECCO study was to (a) determine the prevalence of chronic constipation among adults in Germany and (b) obtain information on their quality-of-life and further medical parameters by a subsequent written survey. The prevalence data from a telephone interview with 15.000 representative adults were recently [4] reported.

The 10-minute interview concluded with sociographic data (education and training, professional status, and family income). Only if constipation had been acknowledged were they asked whether they would provide a postal address for sending a more elaborate questionnaire on this topic.

### 2.1. The Questionnaire

The 8 pages' (37 + 3 items) questionnaire was sent within 2 weeks following the interview. If the questionnaire was not sent back (by a prepaid envelop) within 3 weeks, a reminding letter was sent to the person to ask for completion; a second reminding letter would follow if again no response occurred within three weeks.

The questionnaire was composed of 4 modules (1: General Health; 2: Concurrent Diseases/Medication; 3: Health Care Utilization; 4: Constipation and IBS) and started with some general questions to overall health that contained the items of the Short-Form 12 (SF-12) [20]. Module 2 inquired about the presence of pregnancy and of GI and non-GI diseases (Crohn's disease, ulcerative colitis, GI cancer, celiac disease, and prolapse) and nonintestinal disorders (diabetes, hypothyroidism, stroke, Parkinson's disease, multiple sclerosis) (yes, no) that are frequently associated with constipation and about medication intake of the most frequent drugs (generic and brands) on the German market (contraceptives, beta-blockers, ACE inhibitors, calcium antagonists, diuretics, statins, L-thyroxin, antidiabetics, PPI, pain medication, antidepressants, barbiturates, and sedatives), each to be answered for their frequency (daily, at least twice/week, and less). Similarly, drugs taken for constipation (macrogol, lactulose, sorbitol, bisacodyl, sodium bicarbonate, prucalopride, psyllium, senna, and Glauber's salt) were checked for intake frequency (daily, at least twice/week, and less), efficacy, and side effects. Patients were also asked for changes of nutritional habits because of constipation.

Module 3 inquired about consultation of specialists in the past 12 months, sick-days because of constipation, inpatient treatment, diagnostic procedures performed, and complementary and alternative remedies taken because of CC, including the amounts spent that were not reimbursed by health insurance plans. These data will be reported in a separate paper.

Module 4 contained questions from the Rome III modular questionnaire for IBS and for functional constipation [7].

Final three questions referred to statistics and the willingness to participate in a future follow-up questionnaire study.

The protocol of the study methodology had been reviewed by the Ethics Committee of the Tübingen University Medical School, and the study was conducted in accordance with approved standards for epidemiological research [21].

### 2.2. Statistics

Usually, response rates are reported and used without additionally checking for the representativeness of the cohort. To control for potential self-selection biases in the questionnaire data, we compared individuals with constipation who provided a postal address for sending the questionnaire to those that refused to participate with respect to the sociographic data available from the interview. We also compared those that returned the questionnaire to those that did not, using the same data set. These comparisons were done by groupwise *t*-tests and chi-square tests, where appropriate.

Constipation subsamples were constructed based on predefined criteria: patients with at least one somatic diagnosis and/or more than twice weekly intake of medication for nonconstipation reasons constituted a group labeled “constipation probably associated with a somatic condition” (= putative comorbid constipation) and were excluded from the present analysis; we will report these data in a separate paper.

Among the remaining cases, those meeting Rome III criteria for IBS (based on the algorithm provided by the Rome committee [7]) were labeled IBS-C, those meeting Rome criteria for functional constipation but not IBS-C were called FC-R, and the remaining cases were labeled FC. Subgroups were compared by parametric (ANOVA) and nonparametric tests (chi-square test) where appropriate.

All data are reported as mean ± SD and are unweighted with respect to the initial representative survey. The significance level was set to 0.05 for all tests. Post hoc *t*-tests and chi-square test of subgroup comparisons were not corrected for multiple comparisons but instead only performed when the main (ANOVA, chi-square test) analysis was significant.

## 3. Results

During the telephone survey [4], we identified 2239 individuals acknowledging the presence of constipation symptoms during the preceding 12 months [4]. Of these, 1037 (46.3%) were willing to provide a postal address and were sent the questionnaire. Of these 1037 individuals, 589 returned the questionnaire and provided useable data (56.8%).

### 3.1. Self-Selection Bias Control

We compared the data available from the telephone survey [4] between those constipated individuals that would (*n* = 1037) or would not (*n* = 1202) provide a postal address for sending the questionnaire and between those that did (*n* = 589) or did not (*n* = 448) return the questionnaire (see Table 1).

As can be seen, the two steps (accepting the questionnaire to be sent and returning the questionnaire) generated two distinct self-selection biases in the sample: the first with respect to the severity of the constipation symptoms and the latter with respect to the age and health comorbidity conditions. Patients with more severe constipation symptoms, more acute problems, more medication intake and doctor visits because of constipation, and a higher burden by different constipation symptoms were more willing to provide further information and accepted the questionnaire to be sent.

After they had received the questionnaire, another self-selection bias is apparent: those that returned the questionnaire were on average 10 years older than those that did not respond, and this was associated with a higher percentage being retired, having lower overall life satisfaction, additional (circulation) problems, and a longer duration of constipation. However, second, this self-selection was not based on constipation symptom severity.

### 3.2. IBS-Associated Symptoms in Functional Constipation

Table 2 shows the distribution of the 589 respondents across the IBS and functional constipation-associated symptoms as defined by the Rome criteria.

All symptoms associated with IBS are present to an almost equal extent, and similarly all constipation symptoms are. Also, abdominal pain and discomfort was reported by 4/5, but only a small fraction (approx. 25%) experienced abdominal pain/discomfort at least once a week or more, thus matching the minimal requirement for IBS.

### 3.3. Comorbid Constipation

Among all 589 respondents, 9 women acknowledged being pregnant—they were excluded—leaving 580 data sets to be entered into this analysis.

When asked for concurrent GI and non-GI diagnoses, 245 persons reported one or more diagnoses to be present, and in 314 cases medication was taken at least twice per week, resulting in 366 cases of putative “comorbid constipation” (62.9%). The data of this subsample were excluded from this analysis but will be reported in a separate paper. The 214 remaining cases of “functional constipation” (38.1%) were included and are presented here.

### 3.4. Functional Constipation (IBS-C versus FC-R versus FC)

Of all patients with assumed nonsomatic (“functional”) constipation (*n* = 214), *n* = 64 (11.0% of the total cohort of *n* = 580) met criteria for IBS-C according to the Rome III definition [7], that is, abdominal pain/discomfort at least 3 days per month in addition to constipation, for more than 6 months, not associated with the menstrual cycle, and at least 2 of the following symptoms: symptom improvement with defecation, onset associated with a change in stool frequency, and onset associated with a change in stool form. Of the remaining cases, *n* = 36 (6.2%) met Rome III criteria for functional constipation (FC-R): not meeting the criteria for IBS-C, and at least 2 of the following six symptoms: need for straining, lumpy or hard stools, sensation of incomplete evacuation, sensation of anorectal obstruction, and need for manual maneuvers to facilitate defecation more than occasionally, or less than 3 defecations/week. The remaining 114 cases (19.7%) were classified as “functional constipation” (FC).

Age and sex distribution were similar in all three groups (Table 3) as were social factors (education, income, and job situation).

When asked for their acute health problems, again no major differences occurred between the functional constipation subgroups except that significantly more gastrointestinal symptoms beyond constipation were reported in individuals with IBS-C as compared to FC.

As shown in Table 4, no differences were found for the duration of constipation, doctor visits for constipation during the last 12 months, and medication intake for constipation. While an equal number of the constipated individuals report the presence of hard stools, stool frequency lower than 3/week and straining were more present in the IBS-C and FC-R group as compared to FC.

Current medication intake for constipation was similar for IBS-C (17.2%), FC-R (22.2%), and FC (9.6%) (Table 3), but complementary and alternative medicines (CAM) (homeopathy, acupuncture, and Chinese herbal medicines) for constipation were used by more IBS-C than FC individuals (20.3, 11.1, and 5.2%, resp., *p* = 0.008). A majority of individuals with IBS-C and FC-R claimed to have changed diet to counteract constipation (64.1, 58.3, and 31.6%, resp., *p* < 0.001), and the dietary actions include all measures listed in the questionnaire (more vegetables, more legumes, liquid intake, probiotics, etc.).

**Figure 1:**
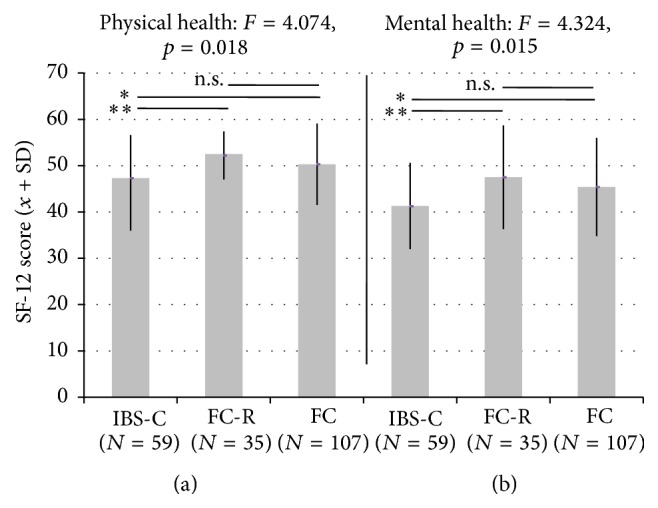
Quality-of-life in groups of constipated participants (individuals with IBS-C, FC-R, and FC, see text for definitions) as measured by the SF-12 (arbitrary units, mean ± SD) in the physical domain (a) and the mental domain (b). *F*-values indicate significance in the between-group ANOVA. “*∗*” indicating significance in post hoc *t*-tests (uncorrected): ^*∗∗∗*^ *p* < 0.001; ^*∗∗*^ *p* < 0.01; ^*∗*^ *p* < 0.05.

Individuals with constipation had similar and lowered QoL on the SF-12 physical health domain in all three groups (IBS-C, FC-R, and FC) compared to population norms of the test, but in IBS-C the scores were also significantly lower in comparison to FC-R and FC, in both the physical health and the mental health domain (Figure 1).

## 4. Discussion

Comparing the responses of individuals reporting to have been constipated in the past 12 months during a telephone interview [4] and after returning a mailed questionnaire, we found only half of the sample to have functional constipation while in the remaining coexistence of somatic disorders (and respective medication intake) may contribute to the constipation symptoms. In search for possible explanations for this effect that has not been described in previous reports, we performed a bias analysis by comparing those that accepted a follow-up questionnaire and those that did not, as well as between those that returned the questionnaire and those that did not. Such post hoc self-selection bias analyses have rarely been reported in previous population surveys on constipation, IBS, and related gastrointestinal issues; they are, however, known from other epidemiological health surveys [22] and assessments of screening programs [23], for example, in the area of pain, cancer, and sexuality [24, 25], and are subject to statistical research on potential correction factors [26]. Usually, response rates are reported and used as if not affecting the representativeness of the cohort. However, as we show here, a strong self-section bias was apparent in our sample: elderly people especially with somatic comorbidity chose to return the questionnaire, while younger ones are underrepresented. We are not reporting the data of this subsample of “somatic constipation” but will do so in our next analysis step and will compare it to other recent reports on comorbidity in constipation [27].

This had also consequences for the remaining and assumed functionally constipated individuals, as it challenges to label these data as representative for Germany, as we did with the data from the telephone survey [4]. However, we have gained a much better characterization and understanding of constipation, either of purely functional origin or with a putative somatic comorbidity.

However, while we may have lost the representativity of our sample of functionally constipated individuals, we have no evidence indicating that the ratio between the three subgroups (IBS-C, FC-R, and FC) has changed; neither is the sex distribution nor the age or any other descriptor any different between them. Thus we can assume that, in Germany, more than half of individuals with functional constipation do not match Rome criteria, neither for IBC-C nor for functional constipation. Whether this holds also true for the other IBS-associated symptoms, specifically diarrhea and alternating bowel habits, cannot be answered with our data, as we did not include patients reporting diarrhea in our survey, neither during telephone interviewing nor with the questionnaire. Hence, we cannot conclude either whether the ratio between IBS-subtypes that has been reported from other countries [28] is maintained in Germany.

Individuals with functional constipation (IBS-C, FC-R, and FC) appear to be similar with respect to most of the social and clinical descriptors assessed in our survey. For example, differential profile has not been observed before. The individual burden of constipation is well established [18, 19, 29, 30], as is its severity association with loss of work productivity [31] and consultation behavior [32]. When patients with IBS-C were compared to patients with FC-R and/or FC, few differences were found: non-IBS patients with painful constipation were younger, were more frequently consulters in comparison to IBS-C [6], had a higher need to strain and more incomplete evacuations [8], and had a higher age at constipation onset and higher mental health compared to IBS-C [9], and both IBS-C and FC patients with pain were significantly more bothered than patients without pain [10]. However, all four surveys did not identify specific and/or homogeneous clinical and QoL profiles as we did in our sample. In our hands, these subgroups did not differ with respect to most measures except for quality-of-life, as measured by SF-12. It appears that the major factor driving the specificity of the QoL profiles is the presence or absence of abdominal pain (Figure 1) because pain (as defined in IBC-C) lowers QoL both in the physical domain and in the emotional domain compared to other functionally constipated individuals. This is in agreement with previous reports indicating abdominal pain being the major determinant of lowered QoL in IBS [10]. When IBS-C and chronically constipated patients not matching IBS criteria were compared to patients with functional dyspepsia (FD) and gastroesophageal reflux disease (GERD) [31], those in which symptoms overlapped reported significantly higher health care utilization—an aspect we will control next in our sample.

One other characteristic of IBS-C patients is evident from our analysis of the telephone survey data (Table 3): IBS-C patients report significantly more general “gastrointestinal symptoms” than individuals with functional constipation not matching Rome criteria (FC). Whether this reflects the fact that these IBS patients suffer from abdominal pain predominantly, or whether this (also) refers to other upper and lower gastrointestinal symptoms, can unfortunately not be explored since neither the telephone survey nor the questionnaire did address such other intestinal symptoms.

Some more limitations of our data analysis need acknowledgement, beyond the self-selection biases discussed above. We also used rather liberal criteria to define “comorbid constipation” based on self-reported diagnoses and/or regular medication intake, the latter with a cut-off of 2 or more days per week. This may have inflated the number of individuals that were assembled in the group with “comorbid constipation” and downsized the number of patients with functional constipation for this analysis, since regular use of a PPI does not necessarily imply functional dyspepsia or gastric ulcer or GERD, and the frequent use of sleeping pills does not necessarily indicate a central or autonomic nervous system disturbance. In some cases, individuals reported intake of diabetic medication but not the diagnosis of diabetes, which my shed light on the comprehension of the questionnaire by some participants. Finally, the presence of a somatic disease does not necessarily indicate that constipation is caused by this disease; it may be incidental comorbidity, and the absence of a somatic condition in those labeled functional constipation does not confirm that a comorbid somatic condition does not exist; epidemiological data relying solely on subjective reports always carry the risk of false information. Therefore, some of the volunteers labeled “comorbid constipation” and excluded for this analysis may instead belong into one of the groups included in the present analysis, and such correction may diminish some of the found differences, although the opposite may happen as well.

## Figures and Tables

**Table 1:** Test for self-selection biases between those providing a post address for sending the questionnaire and those who did not and between those who send back the questionnaire and those who did not. Compared are the data provided during the telephone interview prior to asking for the postal address. Data are unweighted.

	Questionnaire received			Questionnaire returned		
	Yes: *n* = 1037	No: *n* = 1203	Stats^*∗*^	Yes: *n* = 589	No: *n* = 448	Stats^*∗*^
Personal data						
Age (mean, SD)	51.3 ± 0.6	49.9 ± 0.6	n.s.	**55.3 ± 0.7**	**46.0 ± 0.9**	*p* < 0.001
Male : female	352 : 685	453 : 750	n.s.	201 : 388	151 : 297	n.s.
Height (m)	1.69 ± 0.01	1.69 ± 0.01	n.s.	1.69 ± 0.01	1.69 + 0.01	n.s.
Weight (kg)	74.6 ± 0.5	74.9 ± 0.2	n.s.	74.7 ± 0.7	74.5 ± 0.9	n.s.
BMI	26.1 ± 0.2	25.9 ± 0.1	n.s.	26.2 ± 0.2	26.0 ± 0.3	n.s.

Social situation						
Education: secondary^+^	256	284	n.s.	155	101	*p* = 0.010
Full-time/part time (1)	375	449	1/2 versus 3/5 4 excluded: n.s.	195	179	1/2 versus 3/5 4 excluded: *p* = 0.016
Mini job, occasional (2)	72	86		46	27	
Not working, training (3)	186	244		94	92	
Parent time (4)	54	39		18	35	
Retired (5)	347	379		234	113	
Income: >2,500€/mo	284	221		160	123	n.s.

Life satisfaction						
Fully (1)	309	350	1/2 versus 3/4: *p* < 0.001	186	123	1/2 versus 3/4: *p* = 0.007
Rather (2)	531	689		308	223	
Rather not (3)	134	111		68	66	
Not at all (4)	59	49		25	34	

General health						
Very good (1)	111	141	1/2 versus 3/4/5: n.s.	63	48	1/2 versus 3/4/5: n.s.
Good (2)	360	421		217	143	
Satisfactory (3)	305	351		168	137	
Less good (4)	166	176		91	74	
Bad (5)	90	113		48	43	

Health problems						
Sick the last 4 wks: no	705	899	*p* < 0.001	410	295	n.s.
Back pain: yes	684	771	n.s.	390	293	n.s.
Circulation: yes	433	486	n.s.	225	208	*p* = 0.008
Gynacological: yes	106/685	91/750	n.s.	61/388	44/297	n.s.
Urological: yes	145	156	n.s.	82	63	n.s.
Gastrointestinal: yes	330	377	n.s.	189	141	n.s.

Constipation characteristics						
Duration (years)	9.7 ± 0.5	9.2 ± 0.5	n.s.	**11.3 ± 0.7**	**7.8 ± 0.6**	*p* < 0.001
12 months' prevalence	614	762	*p* = 0.042	352	262	n.s.
4 weeks' prevalence	422	441	*p* = 0.052	237	185	n.s.
Acute constipation	196	191	*p* = 0.047	104	92	n.s.
To doctor	240	200	*p* < 0.001	131	109	n.s.
Medication	353	345	*p* = 0.004	194	159	n.s.
<3 stools/week	380	398	*p* = 0.055	199	181	*p* = 0.029
Straining	659	653	*p* < 0.001	388	271	n.s.
Hard stools	764	830	*p* = 0.005	439	325	n.s.

^+^Number with secondary school finished (maturation); ^*∗*^Statistics: *t*-test or chi-square test; n.s.: not significant.

**Table 2:** IBS and constipation symptoms according to the Rome Modular Questionnaire (RMQ) (validated German version). Absolute number of respondents is given. Please note that the sequence of questions was different than in the RMQ because all participants were asked for their constipation symptoms first (52–58, 59), followed by the abdominal pain/discomfort questions (41, 45, 43, 46–50, and 44). Data are unweighted.

RMQ item	Question	Number of respondents (*N* = 589)^+^						
	Frequency of symptoms^*∗*^	0	1		2	3		4
52	Less than 3 stools/week	312	128		72	28		20
53	Hard or lumpy stools	107	262		117	59		7
54	Straining for stools	92	226		132	66		31
55	Feeling of incomplete evacuation	143	222		143	34		12
56	Obstructed defecation	257	193		77	23		7
57	Digital manipulation needed	321	161		41	12		6
58	Problems to relax for evacuation	226	233		59	20		9
46	Pain improved w/defecation	73	95		100	126		96
47	Onset associated w/defecation	264	126		35	39		12
48	Less stools with pain/discomfort	231	148		44	28		13
49	Softer stool with pain/discomfort	202	149		47	56		16
50	Harder stools w/pain/discomfort	165	172		86	53		13
44	Pain/discomfort affecting daily life	251	195		70	17		8
59	Constipation starting > 6 months	No: 203			Yes: 339			
	Frequency^#^	0	1	2	3	4	5	6
41	Abdominal pain/discomfort	149	97	46	129	61	71	17
45	Pain/discomfort for > 6 months	No: 238			Yes: 301			
43	Associated w/menstruation°	No: 211			Yes: 37			

^*∗*^0: never or rarely; 1: sometimes; 2: often; 3: almost always; 4: always; ^#^0: never; 1: <1 day/month; 2: 1 day/month; 3: 2-3 days/month; 4: 1 day/week; 5: >1 day/week; 6: every day; ^+^remainder to 586 are missing/no response; °only women (*N* = 297).

**Table 3:** Sociographic data and health problems and life satisfaction in functionally constipated participants (*n* = 214) in the telephone survey by type of constipation (IBS-C, FC-R, and FC—see text for definitions). Data are unweighted.

Variable name	IBS-C, *N* = 64	FC-R, *N* = 36	FC, *N* = 114	Statistics^#^			
				ANOVA or chi-square	Pairwise post hoc test		
					1-2	1–3	2-3
Personal data							
Age (mean, SD)	44.1 ± 1.6	44.6 ± 2.9	43.2 ± 1.5	n.s.	—	—	—
Male : female	17 : 47	12 : 24	41 : 73	n.s.	—	—	—
Height (m)	1.70 ± 0.01	1.68 ± 0.02	1.70 ± 0.01	n.s.	—	—	—
Weight (kg)	72.0 ± 2.2	65.9 ± 1.9	72.2 ± 1.5	n.s.	—	—	—
BMI	24.7 ± 0.6	23.3 ± 0.5	24.8 ± 0.5	n.s.	—	—	—

Social situation							
Education: secondary^+^	27	9	39	n.s.	—	—	—
Full-time/part time (1)	31	25	56	1/2 versus 3/5 4 excluded: n.s.	—	—	—
Mini job, occasional (2)	9	5	11				
Not working, training (3)	13	3	12				
Parent time (4)	3	0	9				
Retired (5)	8	3	16				
Income: >2,500€/mo	24	17	39	n.s.	—	—	—

Life satisfaction							
Fully (1)	15	14	40	1/2 versus 3/4: *p* = 0.048	*∗*	n.s.	n.s.
Rather (2)	36	21	59				
Rather not (3)	7	1	15				
Not at all (4)	6	0	0				

General health							
Very good (1)	13	6	30	1/2 versus 3/4/5: n.s.	—	—	—
Good (2)	28	20	52				
Satisfactory (3)	12	8	21				
Less good (4)	8	3	9				
Bad (5)	4	0	2				

Health problems							
Sick the last 4 wks: no	48	33	93	n.s.	—	—	—
Back pain: yes	43	22	59	n.s.	—	—	—
Circulation: yes	22	10	21	n.s.	—	*∗*	—
Gynacological: yes	8/47	1/24	12/73	n.s.^+^	—	—	—
Urological: yes	1	4	8	n.s.^+^	—	—	—
Gastrointestinal: yes	29	9	22	*p* < 0.001	n.s.	*∗∗∗*	n.s.

^#^ANOVA: univariate, 3 groups, or 2 × 3 chi-square: in case of significance, pairwise post hoc comparisons; ^+^number with secondary school finished (maturation); ^*∗∗∗*^post hoc testing: ^*∗∗∗*^ *p* < 0.001; ^*∗∗*^ *p* < 0.01; ^*∗*^ *p* < 0.05; ^+^Fisher's Exact Test; n.s.: not significant.

**Table 4:** Clinical data and health care behaviors in the functionally constipated participants (*n* = 214) in the questionnaire by type of constipation (IBS-C, FC-R, and FC—see text for definitions). Data are unweighted.

	IBS-C, *N* = 64	FC-R, *N* = 36	FC, *N* = 114	Statistic^#^			
				ANOVA	Post hoc test		
					1-2	1–3	2-3
Constipation characteristics							
Duration of C (in years)	9.1 ± 1.4	9.6 ± 2.4	8.6 ± 1.2	n.s.	—	—	—
To doctor for C: yes	13	2	15	n.s.	—	—	—
Medication for C: yes	21	6	23	n.s.	—	—	—
<3 stools/w: yes	27	19	32	*p* = 0.004	n.s.	*∗*	*∗∗*
Straining: yes	51	20	65	*p* = 0.006	*∗*	*∗∗*	n.s.
Hard stools: yes	54	28	79	n.s.	—	—	—

Health care behaviors							
Current medication: yes	11	8	11	n.s.	—	—	—
Does it help^*∗*^: yes	11	8	11	n.s.	—	—	—
Side effects^*∗*^: yes	14 (11)	5	11	n.s.	—	—	—
Changed diet: yes	41	21	36	*p* < 0.001	n.s.	*∗∗∗*	*∗∗*
Sick leave for C: yes	4	0	6	n.s.^+^	—	—	—
Inpatient for C: yes	0	0	2	n.s.^+^	—	—	—
CAM for C: yes	13	4	6	*p* = 0.008	n.s.	*∗∗*	n.s.
Currently working: yes	43	28	78	n.s.	—	—	—
Yes: clean WC available^*∗∗*^	37	25	65	n.s.^+^	—	—	—
Yes: WC visit any time^*∗∗*^	12	10	32	n.s.	—	—	—

C: constipation; CAM: complementary and alternative medicine; ^#^ANOVA: univariate, 3 groups, or chi-square: “IBS” versus “functional constipation” (FC-R + FC): only in case of significance, pairwise post hoc comparisons; ^*∗*^only those who take meds; ^*∗∗*^only those who are working; ^*∗∗∗*^post hoc testing: ^*∗∗∗*^ *p* < 0.001; ^*∗∗*^ *p* < 0.01; ^*∗*^ *p* < 0.05; ^+^Fisher's Exact Test; n.s.: not significant.
